# Integral terminal sliding mode-based adaptive driving control method of tracked robots

**DOI:** 10.3389/fpls.2025.1658758

**Published:** 2025-10-21

**Authors:** Zhiqiang Li, Kun Luo, Liang Tao, Yan Zhou

**Affiliations:** School of Mechanical Engineering, Tongling University, Tongling, China

**Keywords:** tracked robot, driving control, adaptive integral terminal sliding mode, uncertain disturbance, field applications

## Abstract

Tracked robots (TR) exhibit significant advantages field applications due to their stability and adaptability to uneven and soft terrains. When the TR operating on soft or uneven terrain, the interaction between the tracks and the ground introduces disturbances, these disturbances leading to challenges in maintaining precise driving control. In this work, we address these issues by proposing an adaptive control strategy for tracked robots. First, the disturbance models are established based on the Bekker pressure-sinkage and Janosi shear theories, enabling a comprehensive understanding of the robot-terrain interaction dynamics. Subsequently, an adaptive integral terminal sliding mode (AITSM) control method is introduced to enhance the robustness and precision of the driving system under complex environmental conditions. Experimental results demonstrate the effectiveness and superior performance of the proposed method in real-world scenarios. This study not only provides a solution for improving the control of tracked robot in outdoor applications but also offers a framework for driving control in a wide range of intelligent field machinery, including agricultural robots, exploration vehicles, and disaster response systems.

## 1 Introduction

The deployment of tracked robots (TR) in field applications has become increasingly prevalent due to their exceptional ability to navigate challenging terrains, such as uneven, soft, or vegetation-covered surfaces. Unlike wheeled robots, TR offer superior traction, stability, and load distribution, making them ideal for tasks in agriculture, exploration, and disaster response (Li et al., 2019; Liu and Liu, 2009). However, their performance in real-world environments is often hindered by the complex dynamic interactions between the tracks and the soil. These interactions introduce disturbances, such as uncertain shear forces and pressure subsidence, which are influenced by factors like soil composition, vegetation density, and external loads (Xie et al., 2024). Such disturbances pose significant challenges to achieving precise driving control, limiting the operational efficiency and reliability of TR in practical applications (Zhang et al., 2022).

Researchers have developed various control systems to achieve good TR performance, employing techniques such as fuzzy control (Hacene et al., 2022; Resende et al., 2013) and nonlinear control (Yan et al., 2022). It is well-established that the aforementioned control approaches, which rely on the robot kinematics model, are primarily applicable to structured environments. However, due to the soft soil and the presence of weeds on the soil surface, the field work environment for TR is quite complex, which is a typical unstructured environment (Xu et al., 2023). The attractive properties of sliding mode control (SMC), namely its ease of execution and robustness to perturbations, make it a favored choice for applications in robotics and mechatronics (Gad et al., 2024; Liu et al., 2020). The application of SMC in robotics is well-documented for addressing challenges like parameter uncertainties and disturbances. For instance, Xi et al. (2022) developed a robust adaptive SMC to achieve accurate and smooth control of robot manipulators under such conditions. Similarly, Liu et al. (2022) designed a novel trajectory tracking controller for a spherical robot by combining controller with a hierarchical SMC scheme, enabling precise velocity tracking across complex terrains. Beyond mobile robots, SMC has also been applied to snake robots for velocity tracking, as demonstrated by Mukherjee et al. (2017). In applications where fast response is critical, such as in TR, the Integral Terminal Sliding Mode Control (ITSMC) variant has been the focus of extensive research (Qin et al., 2024; Su and Zheng, 2020; Sun et al., 2021; Van et al., 2019), due to its enhanced performance. Compared to the traditional SMC with infinite convergence time, ITSMC can stabilize at the equilibrium point within a finite time, ensuring global robustness in the state space from the initial moment, and by using integral sliding mode to design disturbance estimators, continuous control can be achieved, and chattering can be eliminated, while ensuring strong robustness and high accuracy of sliding mode control (Nguyen and Pitakwachara, 2024; Qian et al., 2020; Shen et al., 2023). In (Rahmani et al., 2016), a control scheme based on the fraction integral terminal sliding mode control and adaptive neural network was proposed, which deals with the system model uncertainties and the disturbances to improve the control performance of the manipulator. In (Chiu, 2012), integral TSMC is developed for robust output tracking of uncertain relative-degree-one systems by introducing sign and fractional integral terminal sliding modes, and the control system is forced to start on the terminal sliding hyperplane, so that the reaching time of the sliding modes is eliminated.

Inspired by the aforementioned studies, we propose an adaptive control strategy to address the challenges associated with TR driving control in complex terrains. By leveraging the Bekker pressure-sinkage and Janosi shear theories, we establish disturbance models that capture the robot-terrain interaction dynamics. These models provide a foundation for understanding the effects of soil deformation and shear forces on TR motion. Building on this understanding, we introduce an adaptive integral terminal sliding mode (AITSM) control method, which combines the benefits of adaptive control and terminal sliding mode control to enhance robustness and precision. Experimental validation demonstrates the effectiveness of the proposed method in real-world scenarios, showcasing its ability to maintain precise driving control in challenging environments. This study not only advances the field of TR control but also provides a versatile framework for driving control in a wide range of intelligent field machinery, including agricultural robots (Bai et al., 2023; Wang et al., 2022; Zhang et al., 2022), exploration vehicles, and disaster response systems. By addressing the critical challenges of terrain interaction and disturbance rejection, this work contributes to the broader goal of enhancing the autonomy and reliability of field robots in outdoor applications.

The major contributions can be summarized as follows:

1. Based on Bekker pressure subsidence model and Janosi shear model, the dynamic model of TR is established, to facilitate for the subsequent controller design.
2. An AITSM control scheme is developed to ensure accurate and robust driving control performance of the TR under complex field environment.
3. The designed adaptive controller can well compensate for the shear disturbance caused by pressure subsidence during the actual operation of TR, which further improves its operation stability effectively.
4. Due to the adopted recursive terminal sliding surface, the error state can be well guaranteed both far away from and near the equilibrium without the issue of singularity in a fast convergence rate.

The remainder of this article is constructed below. Section 2 describes the TR system modeling. Section 3 presents the AITSM driving control method with the rigorous stability proof. Section 4 gives real-time experiments on the TR platform and corresponding discussions. Section 5 concludes this paper.

## 2 System modeling

**Figure 1:**
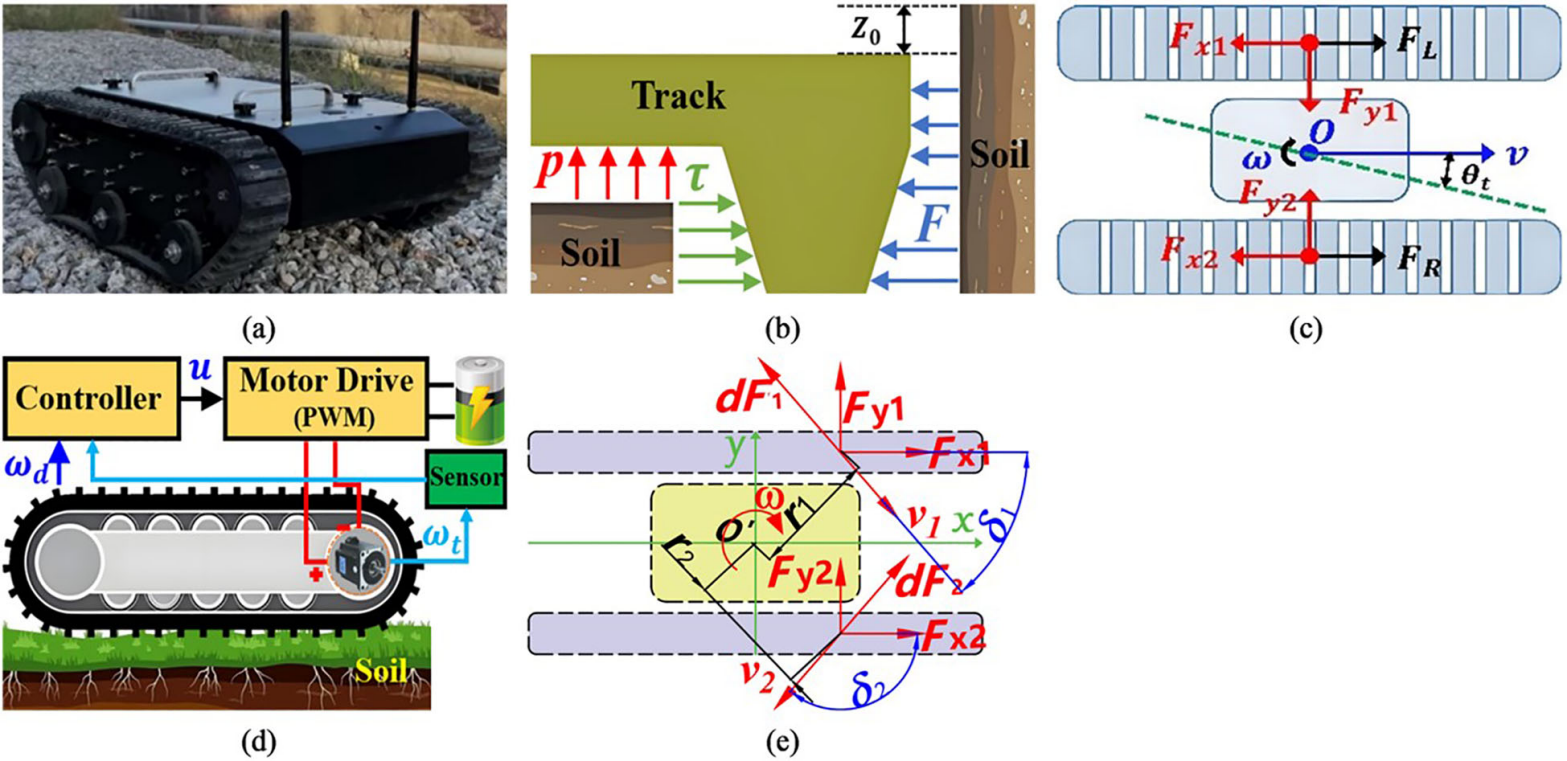
Analysis of the contact characteristics between the chassis and the soil. **(a)** Tracked robot (model no. TR400). **(b)** Dynamical model of contact between soil and TR track. **(c)** Disturbance mechanism of TR. **(d)** Diagram of TR track control. **(e)** Diagram of track steering dynamics on both sides.


**Figure 1a** shows a tracked robot (model no. TR400), which is mainly composed of a control system and a drive system, respectively. Note that, in the field environment, since the soil is soft and sticky, the TR has complex track-ground contact surfaces, which greatly increases the difficulty of driving control. Therefore, the subsidence displacement and sheer force of the TR should be considered, before designing a control method for driving system. The positive pressure between track and ground satisfies the pressure-subsidence model proposed by Bekker (Li et al., 2019), which is shown in **Figure 1b**. Besides, as shown in **Figure 1c**, the relationship between the shear stress of track and the soil deformation satisfies the formula of shear stress and deformation proposed by Janosi (Kayacan et al., 2018). The pressure subsidence and shear can be expressed as Equations 1, 2.

(1) $$p=(k_{c}/b+k_{\emptyset })z_{0}^{n_{k}}$$

(2) $$\begin{array}{c} \tau_{left}=(c+p_{L}\tan\emptyset )(1-exp^{-\frac{j}{k}}),\tau_{right}\\ =(c+p_{R}\tan\emptyset )(1-exp^{-\frac{j}{k}}) \end{array}$$

where p is compressive stress, $\;k_{c}$ is modulus of cohesion of soil deformation, $\;k_{\emptyset }$ is internal friction modulus of soil deformation, $\;z_{0}$ is soil subsidence, $n_{k}$ is soil deformation index, $p_{L}$ and $p_{R}$ are pressure on left and right track unit areas, respectively, $\tau_{left}$ and $\tau_{right}$ are shear force per unit area of left and right track, respectively, c is soil cohesion, ∅ is internal friction angle of soil, *j* is soil shear displacement, *k* is horizontal shear modulus of soil.

As shown in **Figure 1e**, on the soft ground, the shear force between the track and the ground is opposite to the sliding velocity direction of the track. In the **Figure 1e**, $v_{1},\;v\_2$ is Sliding speed of trackpad at any point during steering, $r_{1},r_{2}$ is Distance from any point to geometric center during steering. According to (2), the shear force acting on the grounding section of the track on both sides can be described as follows (Equations 3, 4).


(3) $$dF_{1}=\tau_{left}dA=(c+p_{L}\tan\emptyset )(1-exp^{-\frac{j}{k}})dA$$

(4) $$dF_{2}=\tau_{right}dA=(c+p_{R}\tan\emptyset )(1-exp^{-\frac{j}{k}})dA$$

Where $F_{1},F_{2}$ is Shear force on track plate, *A* is unit area of track contact ground. From (3), (4), the longitudinal forces acting on both sides of the track are as follows (Equation 5).


(5) $$\begin{array}{l} F_{y2}=b\int_{-L/2}^{L/2}(c+p_{R}\tan\emptyset )(1-e^{-\frac{j}{k}})\cos(\pi -\delta_{2})dx\\ F_{y1}=b\int_{-L/2}^{L/2}(c+p_{L}\tan\emptyset )(1-e^{-\frac{j}{k}})\cos(\delta_{1})dx \end{array}$$

Where b is load plate width, $\;\delta_{1}$ and $\delta_{2}$ are angles between sliding velocity at any point of track grounding section and x-axis direction, L is track shoe length. The lateral force acting on both sides of the track is as follows (Equations 6, 7).


(6) $$\begin{array}{l} F_{x2}=b\int_{-L/2}^{L/2}(c+p_{R}\tan\emptyset )(1-e^{-\frac{j}{k}})\sin(\pi -\delta_{2})dx\\ F_{x1}=-b\int_{-L/2}^{L/2}(c+p_{L}\tan\emptyset )(1-e^{-\frac{j}{k}})\sin(\delta_{1})dx \end{array}$$

(7) $$\begin{array}{c} cos(\delta_{1})=x_{1}^{1/2}/{\left(x_{1}^{2}+{\left(B/2\right)}^{2}\right)}^{1/2\;},\;cos(\pi -\delta_{2})\\ =x_{2}^{1/2}/{\left(x_{2}^{2}+{\left(B/2\right)}^{2}\right)}^{1/2\;} \end{array}$$

Where $x_{1},\;x_{2}$ is X-axis abscissa of any point of trackpad.

Remark: To accurately depict the dynamic interaction between the crawler robot and the soft ground and lay the foundation for the subsequent design of high-performance controllers, this paper adopts the classic Bekker pressure-settlement model and the Janosi shear model for mechanical modeling. The advantage of this modeling method lies in its ability to comprehensively describe the core mechanical characteristics of track-soil contact (i.e., compaction resistance and shear thrust) from both vertical and horizontal dimensions. Its parameters have clear physical meanings and serve as a widely verified theoretical basis in the field of ground mechanics. However, this model is rather sensitive to the accuracy of soil parameters and has limitations under heterogeneous soil conditions. For this reason, this paper will design an adaptive control strategy that does not rely on precise model information to estimate and compensate for the lumped uncertainty composed of model uncertainty and external disturbances online, thereby ensuring the robustness of the system in real and complex environments.

?>The schematic diagram of unilateral track control system is shown in **Figure 1d**. Note that the desired velocity and steering angular velocity required for TR to track the desired path are obtained through the Pure-Pursuit path tracking algorithm (PPPT) (Zhang et al., 2019). Take one side crawler driving wheel as an example, the $\omega_{d}$ is desired angular velocity of the driving wheel. The actual angular velocity of the driving wheel, $\omega_{t}$ , is actually measured by the angular velocity sensor of the driving wheel. The voltage control signal u is calculated from the controller, such that the accurate control of the angular velocity of the driving wheel can be realized. The track is driven by the drive motor through the reducer to drive the drive wheel. The system dynamics of the unilateral track system of TR and the DC motor are given by (Equations 8–11).


(8) $$J_{L}\dot{\omega_{t}}=\tau -B_{L}\omega_{t}-\tau_{F}$$

(9) $$J_{m}\dot{\omega_{m}}=\tau_{a}-B_{m}\omega_{m}-\tau_{m}$$

(10) $$\tau_{a}=k_{t}((u-k_{e}\omega_{m})/R_{d})$$

(11) $$\tau =i\varsigma \tau_{m}$$

Where $J_{L}$ and $J_{m}$ are the moments of inertia of the unilateral track system and motor, respectively, $B_{L}$ and $B_{m}$ are the viscous damping coefficients of the unilateral track system and motor, respectively, $\omega_{m}$ is the motor angular velocity, satisfying $\omega_{m}$ = $i\omega_{t}$ with i defined as the gear ratio, $k_{t}$ and $k_{e}$ are the constants of motor torque and electromotive force, respectively, $\tau_{F}$ is the load torque caused by external disturbance such as $F_{xi}$ and $F_{yi}$ ( i=1,2 ), $R_{d}$ is the total resistance of the armature circuit, u is the control input voltage, $\tau_{a}$ is the motor torque, $\tau_{m}$ is the torque transmitted from the motor to the reducer, τ is drive wheel torque, and ς is torque transmission loss coefficient. Using (9)-(11) into (8) by eliminating $\omega_{m}$ , the dynamics of the unilateral track system can be simplified as (Equation 12).


(12) $$\begin{array}{c} (J_{L}+i^{2}\varsigma J_{m})\dot{\omega_{t}}+(B_{L}+i^{2}\varsigma B_{m}+(i^{2}\varsigma k_{t}k_{e})/R_{d})\omega_{t}+\tau_{F}\\ =((i\varsigma k_{t})/R_{d})u \end{array}$$

To facilitate the further controller design, (12) is reformulated as (Equation 13).


(13) $$m\dot{\omega_{t}}+n\omega_{t}+d_{l}=u$$

Where $m=(J_{L}+i^{2}\varsigma J_{m})R_{d}/i\varsigma k_{t}$ , $n=(B_{L}+i^{2}\varsigma B_{m}+i^{2}\varsigma k_{t}k_{e}/R_{d})R_{d}/i\varsigma k_{t}$ , $d_{l}=\tau_{F}R_{d}/i\varsigma k_{t}$ . In this paper, we consider the following parametric variations in (13) as follows (Equations 14, 15).


(14) $$m=m_{0}+\Delta m$$

(15) $$n=n_{0}+\Delta n$$

Where $m_{0}=0.043\;kg\cdot m^{2}$ , $n_{0}=1.025$ N·m·s/rad are the nominal values and Δm , Δn are their uncertainties, respectively. Note that, $m_{0}$ and $n_{0}$ are the nominal parameters of the system, and their values are determined based on the specific physical parameters of the motor and mechanical structure of the TR400 experimental platform. The tracking error of the angular velocity is defined as (Equation 16).


(16) $$e(t)=\omega_{t}(t)-\omega_{d}(t)$$

Where $\omega_{d}(t)$ should be once differentiable as $\dot{\omega_{d}}(t)$ . The error dynamics can then be obtained from (13) and (16) as follows (Equation 17).


(17) $$\dot{e}(t)=(u(t)-n_{0}\omega_{t}(t))/m_{0}-D_{lum}(t)-\dot{\omega_{d}}(t)$$

Where $D_{lum}(t)=[\Delta m\dot{\omega_{t}}(t)+\Delta n\omega_{t}(t)+d_{l}(t)]/m_{0}$ represents the lumped uncertainty in the error dynamics.

In terms of the bound derivation of the lumped uncertainty. if the closed-loop control u is designed to satisfy the following polynomial-type upper bound as (Equation 18).


(18) $$\left|u(t)|<\zeta_{0}+\zeta_{1}|\omega_{t}(t)\right|$$

Where $\zeta_{i}\;(i=0,1)$ are positive constants, then the lumped uncertainty in (17) will be bounded as (Equation 19).


(19) $$\left|D_{lum}(t)|<d(t\right)$$

Where d(t) is defined as (Equation 20).


(20) $$d(t)=D_{0}+D_{1}|\omega_{t}(t)|$$

with $D_{i}\;(i=0,1)$ being positive constants.

## 3 Design of controller

In this part, an AITSM driving control scheme is developed for the unilateral track system of TR with uncertain dynamics. A precise position tracking performance with finite-time convergence and good robustness can be well ensured, also, the lumped uncertainty bound and the sliding mode parameters are all online updated by the designed adaptive laws, such that the requirements of obtaining the bound information in the controller are successfully eliminated.

### 3.1 Controller design

Firstly, a recursive integral terminal sliding variable is defined as (Equation 21).


(21) $$s(t)=e(t)+\hat{\lambda }(t)e_{I}(t)$$

Where the sliding parameter $\hat{\lambda }(t)$ is to be adaptively adjusted by the following adaptive law, the fast nonsingular terminal sliding function $e_{I}(t)$ is given by Equation 22.


(22) $$\dot{e_{I}}(t)=k_{1}|e(t){|}^{\mu_{1}}sign[e(t)]+k_{2}|e(t){|}^{\mu_{2}}sign[e(t\;)]$$

Where $k_{1}$ and $k_{2}$ are two positive constants, $\mu_{1}>0$ and $\mu_{2}>0$ . It can be clearly observed from (21) that if an initial condition of the integral term $e_{I}(0)$ is chosen as $e_{I}(0)=-{\hat{\lambda }}^{-1}(0)e(0)$ , the sliding variable s(t) will be initially starting from the sliding surface s(0)=0 . Following this nice feature, the reaching phase of the sliding mode control system can be eliminated, which further enhances the fast response and robustness.

The proposed control law u(t) is of the following form (Equation 23).


(23) $$u(t)=u_{eq}(t)+u_{sw}(t)+u_{re}(t)$$

where u_eq(t), u_sw(t), u_re(t) are defined as Equations 24–26.


(24) $$\begin{array}{c} u_{eq}(t)=n_{0}\omega_{t}(t)+m_{0}\omega_{d}(t)\\ -m_{0}\hat{\lambda }(t)\{k_{1}|e(t){|}^{\mu_{1}}sign[e(t)]+k_{2}|e(t){|}^{\mu_{2}}sign[e(t)]\} \end{array}$$

(25) $$u_{sw}(t)=\hat{d}(t)sign[e(t)]$$

(26) $$u_{re}(t)=-\xi_{1}s(t)-\xi_{2}|s(t){|}^{\mu_{3}}sign[s(t)]$$

Where the reaching control parameters $\xi_{1}>0$ , $\xi_{2}>0$ and $0<\mu_{3}<1$ , the parameter $\hat{d}(t)={\hat{D}}_{0}(t)+{\hat{D}}_{1}(t)|\omega_{t}(t)|$ is the estimated value of d(t) , where ${\hat{D}}_{0}(t)$ and ${\hat{D}}_{1}(t)$ together with $\hat{\lambda }(t)$ are updated by the following adaptive laws (Equations 27–29).


(27) $${\dot{\hat{D}}}_{0}=\eta_{0}|s(t)|$$

(28) $${\dot{\hat{D}}}_{1}=\eta_{1}|s(t)||\omega_{t}(t)|$$

(29) $$\dot{\hat{\lambda }}=-\eta_{2}e_{I}(t)s(t)$$

**Figure 2:**
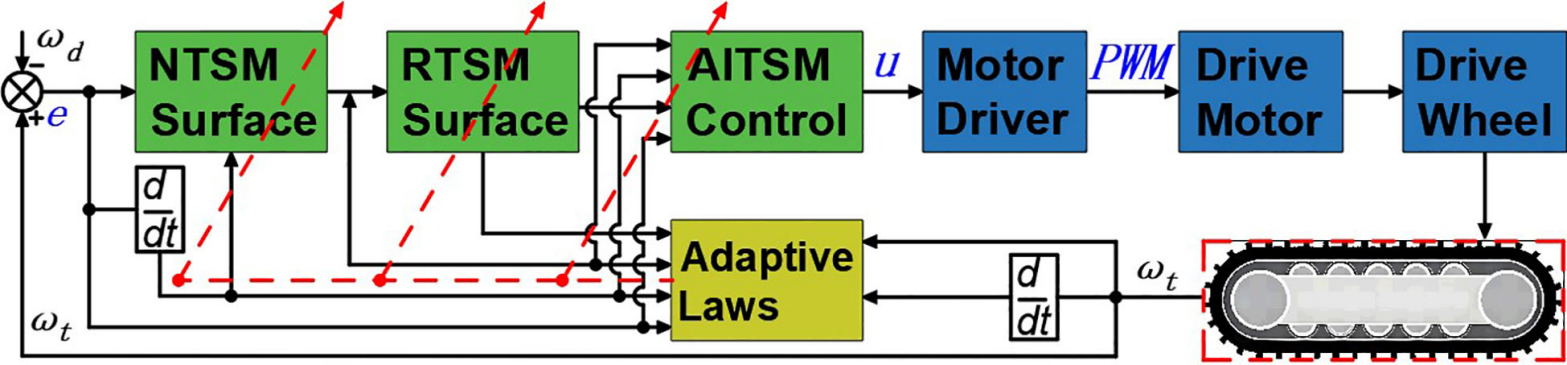
Block diagram proposed AITSM controller.

Where $\eta_{i}(i=0,1,2)$ are positive adaptation rates. The block diagram of the proposed AITSM controller is shown in **Figure 2**, where the right track control system is the same as the left one.

In the following context, for the conciseness of the paper, the notions of time for all given variables are omitted. And for the concise of the paper, the notations of time for all variables are thus omitted in the rest of the paper. In practice, due to the measurement noise, certain deviations of the sliding variables from the sliding mode surface always occur, which causes the estimated bounds to continuously increase and experience undesired parameter bursting. The estimated gains may finally drift to undesired values. To tackle this issue, we use the Equations 30 and 31 dead-zone modification mechanism in the adaptation process (Mathew and Hiremath, 2018; Wang et al., 2016):


(30) $${\dot{\hat{D}}}_{0}=\{\begin{array}{ll} \eta_{0}|s| & for\;|s|\geq \epsilon_{e1}\\ 0 & for\;|s|<\epsilon_{e1} \end{array}$$

(31) $$\dot{\hat{\lambda }}=\{\begin{array}{ll} -\eta_{2}e_{I}s & \text{for }|s|\geq \epsilon_{e2}\\ 0 & \text{for }|s|<\epsilon_{e2} \end{array}$$

Where: $\epsilon_{e1}>0$ , $\epsilon_{e2}>0$ , are the designed positive threshold values and chosen as $\epsilon_{e1}=2.3$ , $\epsilon_{e2}=0.002$ . Note that, the thresholds $\epsilon_{e1}$ and $\epsilon_{e2}$ are based on the assessment of the measurement noise level of the system and are tuned through a series of simulation experiments. The aim is to effectively suppress the parameter drift caused by measurement noise while ensuring adaptability.

### 3.2 Stability proof

Before the stability proof of the proposed control, the following Lemma is given in advance with the corresponding proof given in.

Lemma 1: Given the unilateral track system of TR in (13) and the control law in (25) ${\hat{D}}_{i}\;(i=0,1)$ will be always bounded, i.e., there exists positive numbers $D_{i}\;(i=0,1)$ , such that the following inequality Equation 32 always hold:


(32) $${\hat{D}}_{i}\leq D_{i}\;(i=0,1)$$

Theorem 1: Consider the unilateral track system TR model in (13) with parametric variations in (14)-(15). The closed-loop error dynamics in (17) converges to zero in a finite time under the control law designed in (23).

Proof: First, we give the first derivative of the sliding variable s in (21) as follows (Equation 33).


(33) $$\begin{array}{c} \dot{s}=\dot{e}+\dot{\hat{\lambda }}e_{I}+\hat{\lambda }\dot{e_{I}}\\ =\hat{d}sign(e)-\xi_{1}s-\xi_{2}|s{|}^{\mu_{3}}sign(s)+\dot{\hat{\lambda }}e_{I}-D_{lum} \end{array}$$

Next, considering the following Lyapunov function candidate (Equation 34).


(34) $$V=S^{2}/2+(\rho_{0^{-1}}{\tilde{D}}_{0}^{2})/2+(\rho_{1^{-1}}{\tilde{D}}_{1}^{2})/2$$

and differentiating *V* with respect to time, we have Equation 35.


(35) $$\begin{array}{l} \dot{V}=s\dot{s}+\rho_{0^{-1}}{\tilde{D}}_{0}{\dot{\hat{D}}}_{0}+\rho_{1^{-1}}{\tilde{D}}_{1}{\dot{\hat{D}}}_{1}\\ \;=s\left(\hat{d}sign\left(e\right)-\xi_{1}s-\xi_{2}|s{|}^{\mu_{3}}sign\left(s\right)+\dot{\hat{\lambda }}e_{I}-D_{lum}\right)\\ +\rho_{0^{-1}}{\tilde{D}}_{0}{\dot{\hat{D}}}_{0}+\rho_{1^{-1}}{\tilde{D}}_{1}{\dot{\hat{D}}}_{1}\\ \;=\hat{d}\left(t\right)|s|-\xi_{1}s^{2}-\xi_{2}|s{|}^{\mu_{3}+1}-sD_{lum}+s\dot{\hat{\lambda }}e_{I}+\rho_{0^{-1}}{\tilde{D}}_{0}{\dot{\hat{D}}}_{0}\\ +\rho_{1^{-1}}{\tilde{D}}_{1}{\dot{\hat{D}}}_{1}\\ \;=\hat{d}\left(t\right)|s|-\xi_{1}s^{2}-\xi_{2}|s{|}^{\mu_{3}+1}-sD_{lum}-\eta_{2}e_{I}^{2}s^{2}+\rho_{0^{-1}}{\tilde{D}}_{0}{\dot{\hat{D}}}_{0}\\ +\rho_{1^{-1}}{\tilde{D}}_{1}{\dot{\hat{D}}}_{1}\\ \;\leq -sD_{lum}+\hat{d}\left(t\right)|s|-\xi_{1}s^{2}-\xi_{2}|s{|}^{\mu_{3}+1}+\;\rho_{0^{-1}}{\tilde{D}}_{0}{\dot{\hat{D}}}_{0}\\ +\rho_{1^{-1}}{\tilde{D}}_{1}{\dot{\hat{D}}}_{1}\\ \;=-|s|\left(D_{0}+D_{1}|\omega_{t}|\right)+\left({\hat{D}}_{0}+{\hat{D}}_{1}|\omega_{t}|\right)|s|-\xi_{1}s^{2}-\xi_{2}|s{|}^{\mu_{3}+1}\\ +\;\rho_{0^{-1}}{\tilde{D}}_{0}{\dot{\hat{D}}}_{0}+\rho_{1^{-1}}{\tilde{D}}_{1}{\dot{\hat{D}}}_{1}\\ \;=|s|\left({\tilde{D}}_{0}+{\tilde{D}}_{1}|\omega_{t}|\right)-\xi_{1}s^{2}-\xi_{2}|s{|}^{\mu_{3}+1}+\;\rho_{0^{-1}}{\tilde{D}}_{0}{\dot{\hat{D}}}_{0}\\ +\rho_{1^{-1}}{\tilde{D}}_{1}{\dot{\hat{D}}}_{1}\\ \;=|s|\left({\tilde{D}}_{0}+{\tilde{D}}_{1}|\omega_{t}|\right)-\xi_{1}s^{2}-\xi_{2}|s{|}^{\mu_{3}+1}+\rho_{0^{-1}}{\tilde{D}}_{0}\eta_{0}|s|\\ +\rho_{1^{-1}}{\tilde{D}}_{1}\eta_{1}|s||\omega_{t}|\\ \;=|s|{\tilde{D}}_{0}\left(\rho_{0}^{-1}\eta_{0}+1\right)+|s||\omega_{t}|\left(\rho_{1}^{-1}\eta_{1}+1\right){\tilde{D}}_{1}-\xi_{1}s^{2}-\xi_{2}|s{|}^{\mu_{3}+1} \end{array}$$

since, $\rho_{0}^{-1}\eta_{0}+1>0$ , $\rho_{1}^{-1}\eta_{1}+1>0$ , ${\tilde{D}}_{i}={\hat{D}}_{i}-D_{i}<0$ , we have Equation 36.


(36) $$\begin{array}{l} \dot{V}=-|s||{\tilde{D}}_{0}||\rho_{0}^{-1}\eta_{0}+1|-|s||\omega_{t}||{\tilde{D}}_{1}||\rho_{1}^{-1}\eta_{1}+1|\;-|s|(\xi_{1}|s|+\xi_{2}|s{|}^{\mu_{3}})\\ \leq -\Gamma_{s}\sqrt{2}\sqrt{2^{-1}}|s|-\Gamma_{0}\sqrt{2\rho_{0}}\sqrt{2^{-1}\rho_{1}^{-1}}|{\tilde{D}}_{0}|-\Gamma_{1}\sqrt{2\rho_{1}}\sqrt{2^{-1}\rho_{1}^{-1}}|{\tilde{D}}_{1}|\\ \leq -\Omega (\sqrt{2^{-1}}|s|+\sqrt{2^{-1}\rho_{0}^{-1}}|{\tilde{D}}_{0}|+\sqrt{2^{-1}\rho_{1}^{-1}}|{\tilde{D}}_{1}|)\\ \leq -\Omega V^{\frac{1}{2}} \end{array}$$

Where $\Omega =min(\Gamma_{s}\sqrt{2},\;\Gamma_{0}\sqrt{2\rho_{0}},\;\Gamma_{1}\sqrt{2\rho_{1}})$ , $\Gamma_{s}=\xi_{1}|s|+\xi_{2}|s{|}^{\mu_{3}}$ , $\Gamma_{0}=|s||\rho_{0}^{-1}\eta_{0}+1|$ , $\Gamma_{1}=|s||\rho_{1}^{-1}\eta_{1}+1|$ . According to Lemma 1 and inequality (36), since the constants $\rho_{i}\;(i=0,1)$ always exist to satisfy $\rho_{i}^{-1}\eta_{i}>-1$ , it can be verified that Ω>0 and thus the recursive sliding variable s can have a finite-time zero-convergence. Thus, the finite-time convergence of the sliding variable e is then achieved in the sliding mode s=0 . Finally, after s=0 is fulfilled and maintained, the output tracking error of the angular velocity e will correspondingly converge to zero within a finite time.

This completes the whole proof.

## 4 Experimental study

### 4.1 Experimental configurations

**Figure 3:**
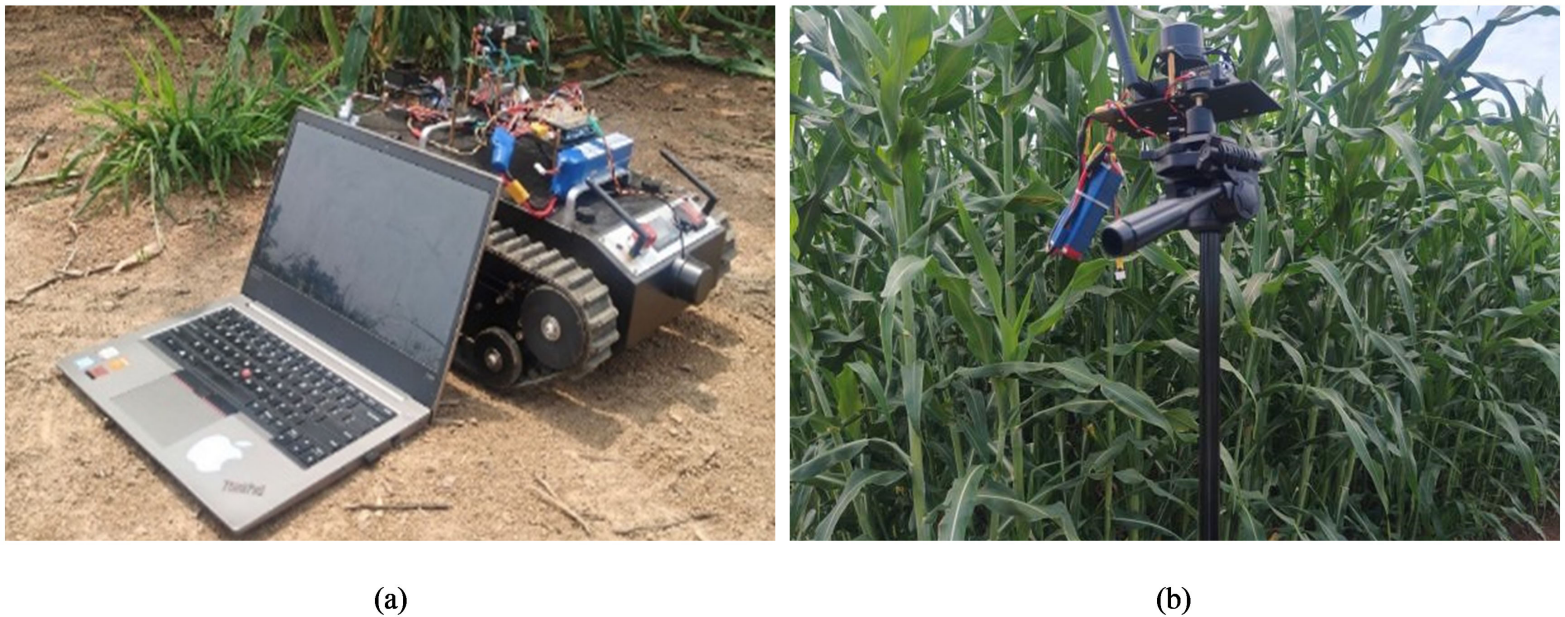
Field test environment and platform. **(a)** TR400 field test platform. **(b)** Navigation system base station.

To validate the effectiveness and practical performance of the proposed Adaptive Integral Terminal Sliding Mode (AITSM) control method, comprehensive field tests were conducted using TR platform. The experimental setup employs Mission Planner as the navigation upper computer system, which automates the ground control station operations and enables autonomous TR navigation through its advanced task planning module. The field test environment and platform are shown in **Figure 3**. For rigorous performance benchmarking, the proposed AITSM controller is compared against two conventional approaches, a traditional Sliding Mode Controller (SMC) and a Proportional Integral Derivative (PID) controller (Li et al., 2019). All controller parameters have been systematically tuned and are comprehensively documented in **Table 1** to ensure fair comparison conditions. The TR’s onboard sensors provide real-time state feedback, while the control algorithms execute at 100Hz sampling frequency.

**Table 1:** Controller parameter values.

Controllers	Parameter values
AITSM	$k_{1}=k_{2}=20$ , $\dot{\hat{\lambda }}(0)=20$ , $\eta_{0}=300$ , $\eta_{1}=60$ , $\eta_{2}=300$ , $\mu_{1}=2.5$ , $\mu_{2}=0.4$ , $\mu_{3}=0.25$ , $\xi_{1}=80$ ,
SMC	$K_{SMC}=3.2$ , $\eta_{SMC}=0.51$
PID	$K_{p}=8.2$ , $K_{i}=1.3$ , $K_{d}=0.1$

The PID control law is Equation 37.


(37) $$u_{PID}=K_{P}e+\frac{1}{K_{i}}\int edt+K_{d}\dot{e}$$

The traditional SMC control is as Equations 38, 39. 


(38) $$u_{SMC}=\omega_{d}-K_{smc}s_{SMC}-\eta_{SMC}sign(s_{SMC})$$

(39) $$s_{SMC}=\omega_{t}-\omega_{d}$$

where: $K_{p}$ is proportional gain of PID controller, $K_{i}$ is integral gain of PID controller, $K_{d}$ is derivative gain of PID controller. $S_{SMC}$ is sliding mode surface of SMC, $K_{smc}$ is sliding mode surface gain of SMC, $\eta_{SMC}$ is switching gain of SMC.

### 4.2 Field test study

#### 4.2.1 Case 1: L-shaped path tracking and robustness

**Figure 4:**
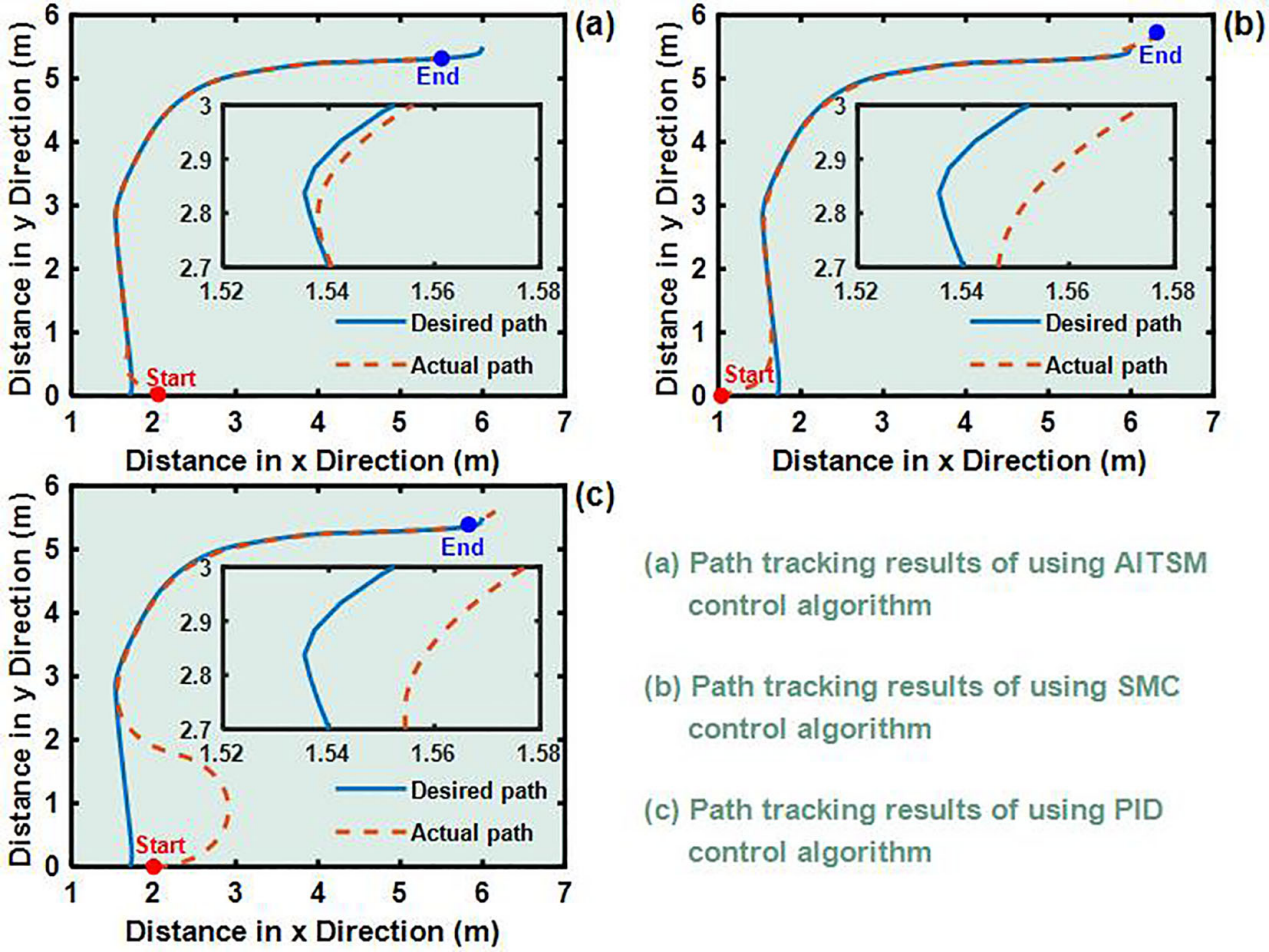
L-shaped path tracking test results **(a)** Path tracking results of using AITSM control algorithm. **(b)** Path tracking results of using SMC control algorithm. **(c)** Path tracking results of using PID control algorithm..

**Figure 5:**
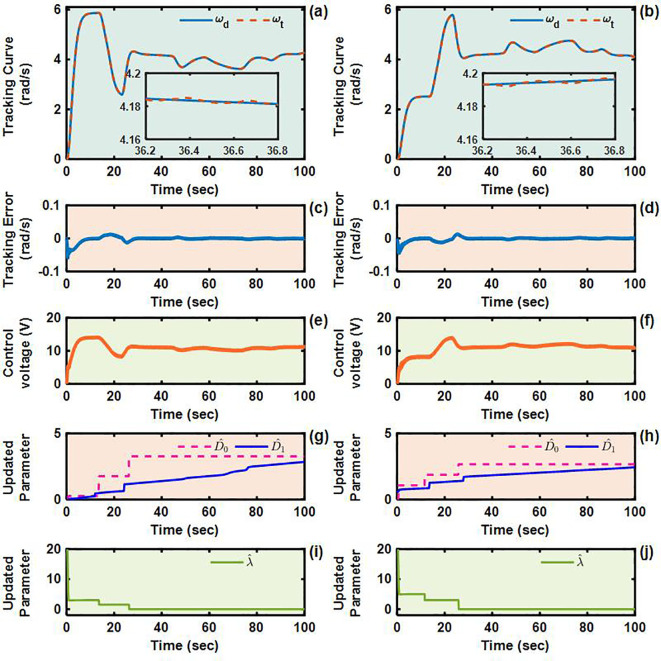
Angular velocity tracking responses of AITSM control for left and right driving wheels of TR (L-shaped path). **(a, b)** are the tracking curves of the angular velocity of left and right driving wheels. **(c)** and **(d)** are tracking errors of left and right driving wheels. **(e, f)** are control voltages of left and right driving wheels. **(g–j)** are updated parameters of left and right driving wheels.

**Figure 6:**
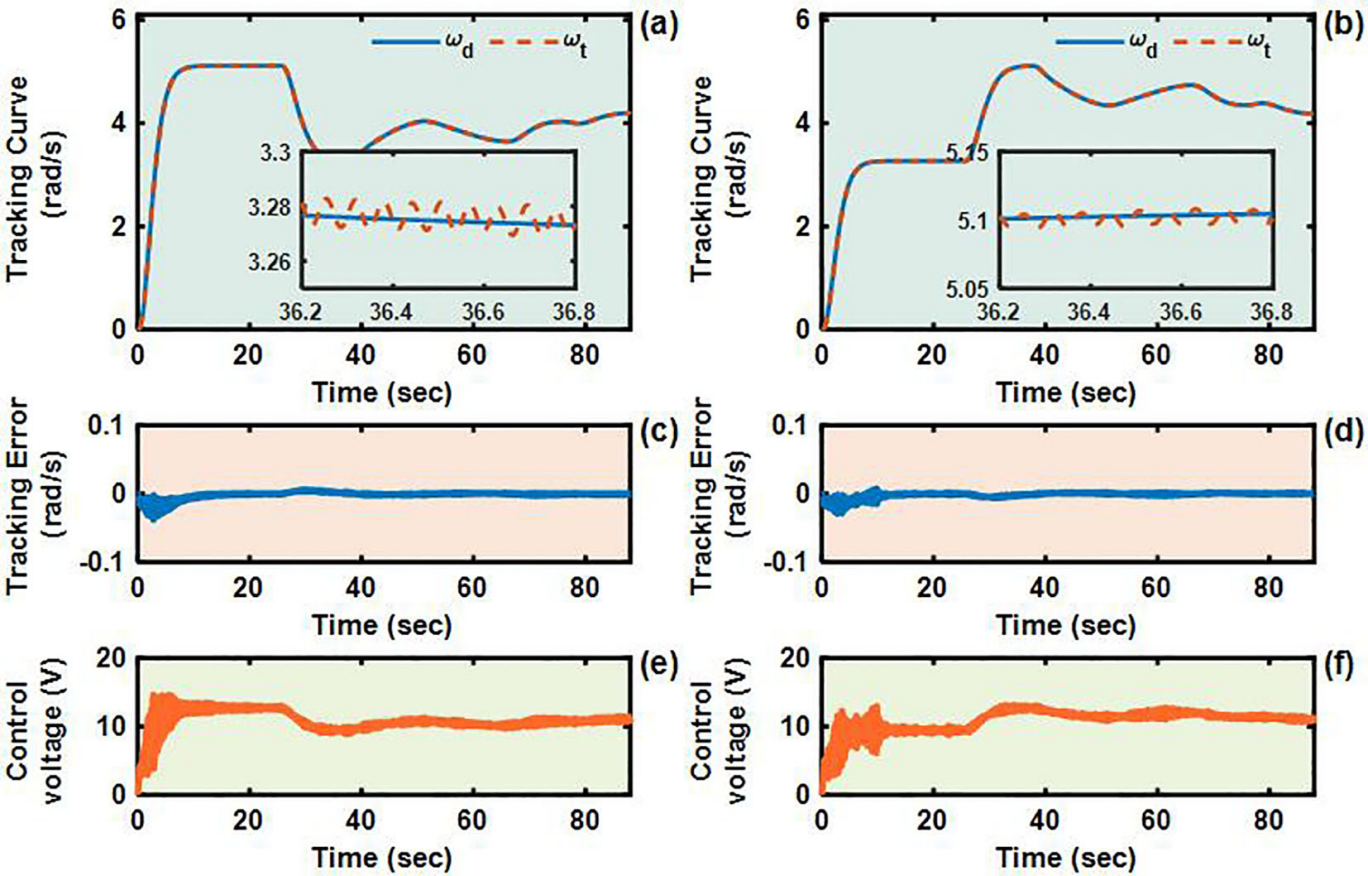
Angular velocity tracking responses of SMC control for left and right driving wheels of TR (L-shaped path). **(a, b)** are the tracking curves of the angular velocity of left and right driving wheels. **(c, d)** are tracking errors of left and right driving wheels. **(e, f)** are control voltages of left and right driving wheels.

**Figure 7:**
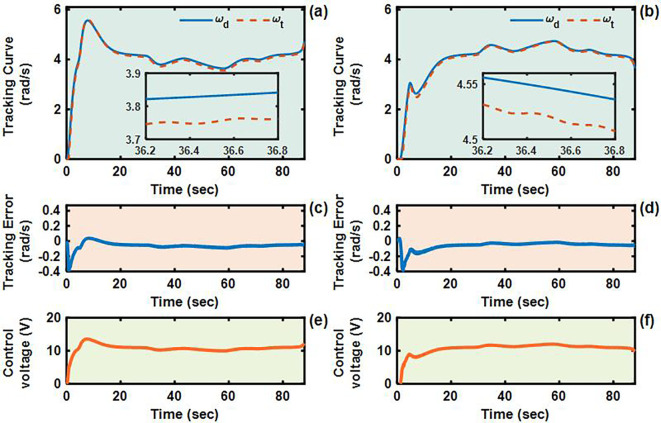
Angular velocity tracking responses of PID control for left and right driving wheels of TR (L-shaped path). **(a, b)** are the tracking curves of the angular velocity of left and right driving wheels. **(c, d)** are tracking errors of left and right driving wheels. **(e, f)** are control voltages of left and right driving wheels.

To validate the control performance of the proposed Adaptive Integral Terminal Sliding Mode (AITSM) controller under realistic operating conditions, we conducted comprehensive experimental evaluations using an L-shaped path tracking scenario that combines straight-line motion with sharp left turns, a common maneuver required in field operations. As shown in **Figure 4**, we can clearly see that the designed controller achieves the best path following responses, followed by the SMC as well as PID controllers. It indicates that the TR with the proposed control is relatively stable during driving, particularly during the critical transition phase between straight-line motion and turning, where the PID controller shows substantial tracking errors. This enhanced performance is particularly critical for field robotic operations where precise navigation through challenging terrain is essential to ensure mission success and operational safety. Further examination of the drive motor responses in **Figures 5**–**7** provides deeper insights into the controllers’ dynamic performance, **Figures 5**-**7a, b** and **Figures 5**-**7c-d** showing that while both the AITSM and SMC controllers maintain satisfactory angular velocity tracking, the AITSM achieves significantly lower average tracking errors of 0.023 rad/s and 0.025 rad/s for the left and right wheels respectively, compared to 0.033 rad/s and 0.027 rad/s for SMC and substantially higher errors of 0.094 rad/s and 0.086 rad/s for PID control. More importantly, the angular velocity tracking result of the SMC controller shows a more obvious chattering phenomenon. This is because the SMC forces the system state to move along the sliding surface through high-frequency switching control signals, as shown in **Figures 5e–f**. This chattering phenomenon poses a greater threat to the control results of the motor and the driving stability of the robot. In contrast, the AITSM controller’s innovative architecture, which combines equivalent control $u_{eq}$ for disturbance compensation with adaptive switching terms $u_{sw}$ for residual uncertainty handling, achieves robust performance while dramatically reducing control signal chattering, as clearly evidenced in **Figures 5g–j**. This dual-mechanism approach allows the AITSM controller to maintain excellent tracking precision, with 30.3% and 7.4% lower errors than SMC for left and right wheels respectively, and 75.5% and 70.9% improvement over PID, while ensuring smooth actuator operation, making it particularly suitable for field applications where prolonged operation and equipment longevity are critical concerns. The superior performance of the AITSM controller stems from its ability to adaptively adjust control parameters in response to varying terrain conditions and system uncertainties, a feature lacking in both conventional SMC and PID approaches. Furthermore, the experimental data confirms that the AITSM controller’s disturbance rejection capability remains effective throughout the entire operating range, from steady-state straight-line motion to dynamic turning maneuvers, without exhibiting the performance degradation seen in PID control during transient conditions or the high-frequency oscillations characteristic of SMC implementations.

#### 4.2.2 Case 2: U-shaped path tracking and robustness

**Figure 8:**
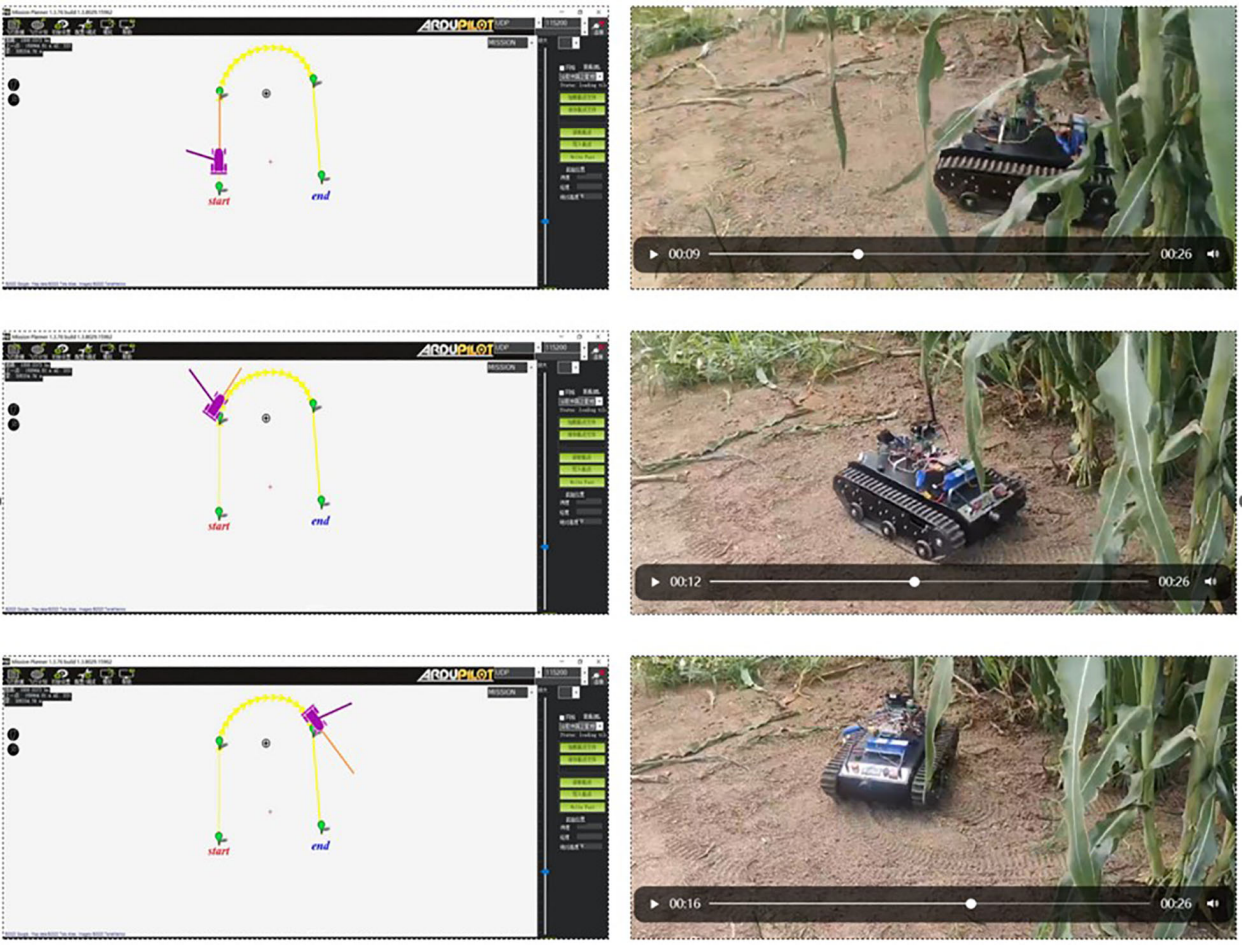
The actual driving conditions of the TR during the U-shaped path tracking test.s.

**Figure 9:**
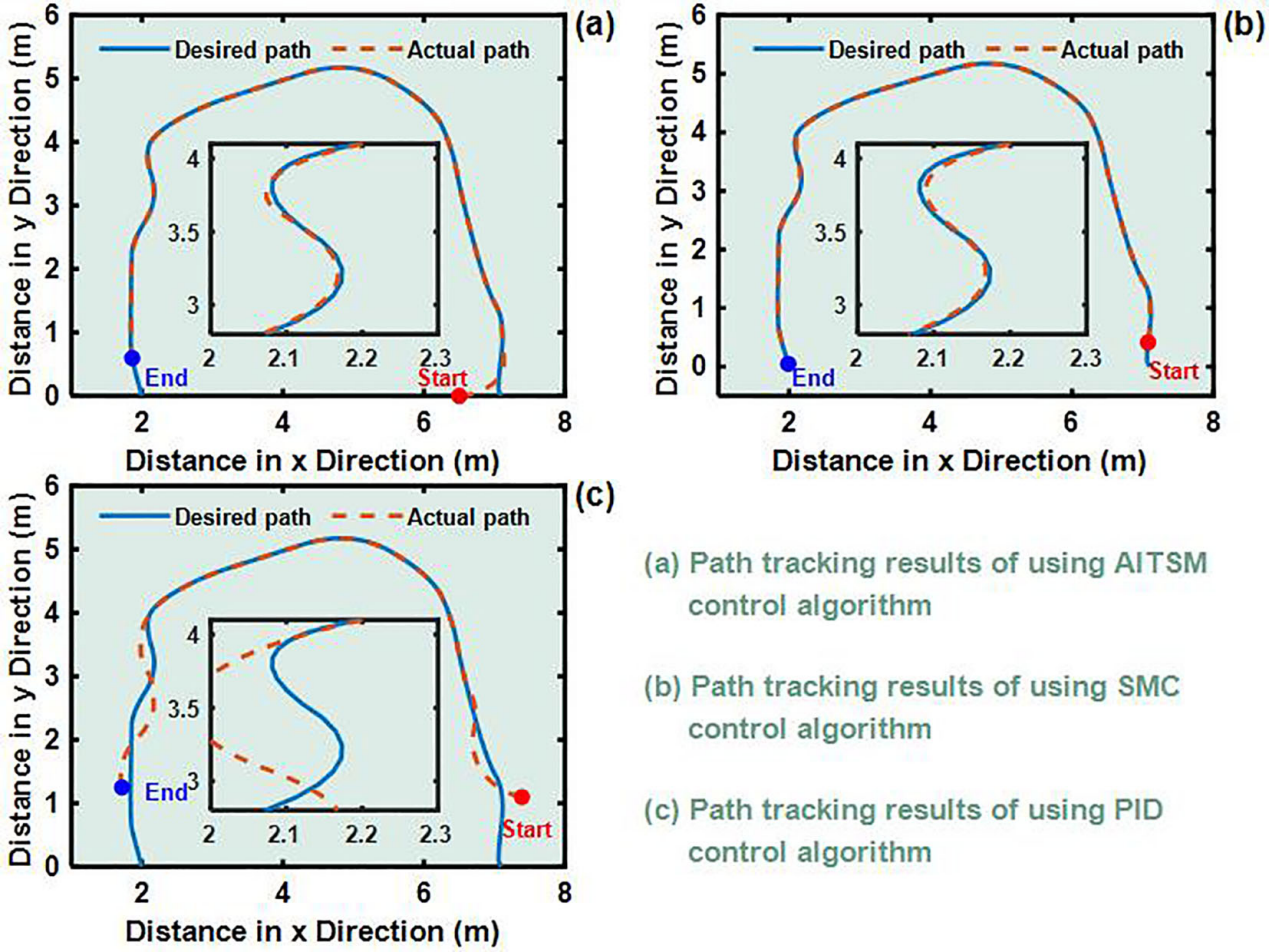
U-shaped path tracking test results. **(a)** Path tracking results of using AITSM control algorithm. **(b)** Path tracking results of using SMC control algorithm. **(c)** Path tracking results of using PID control algorithm..

**Figure 10:**
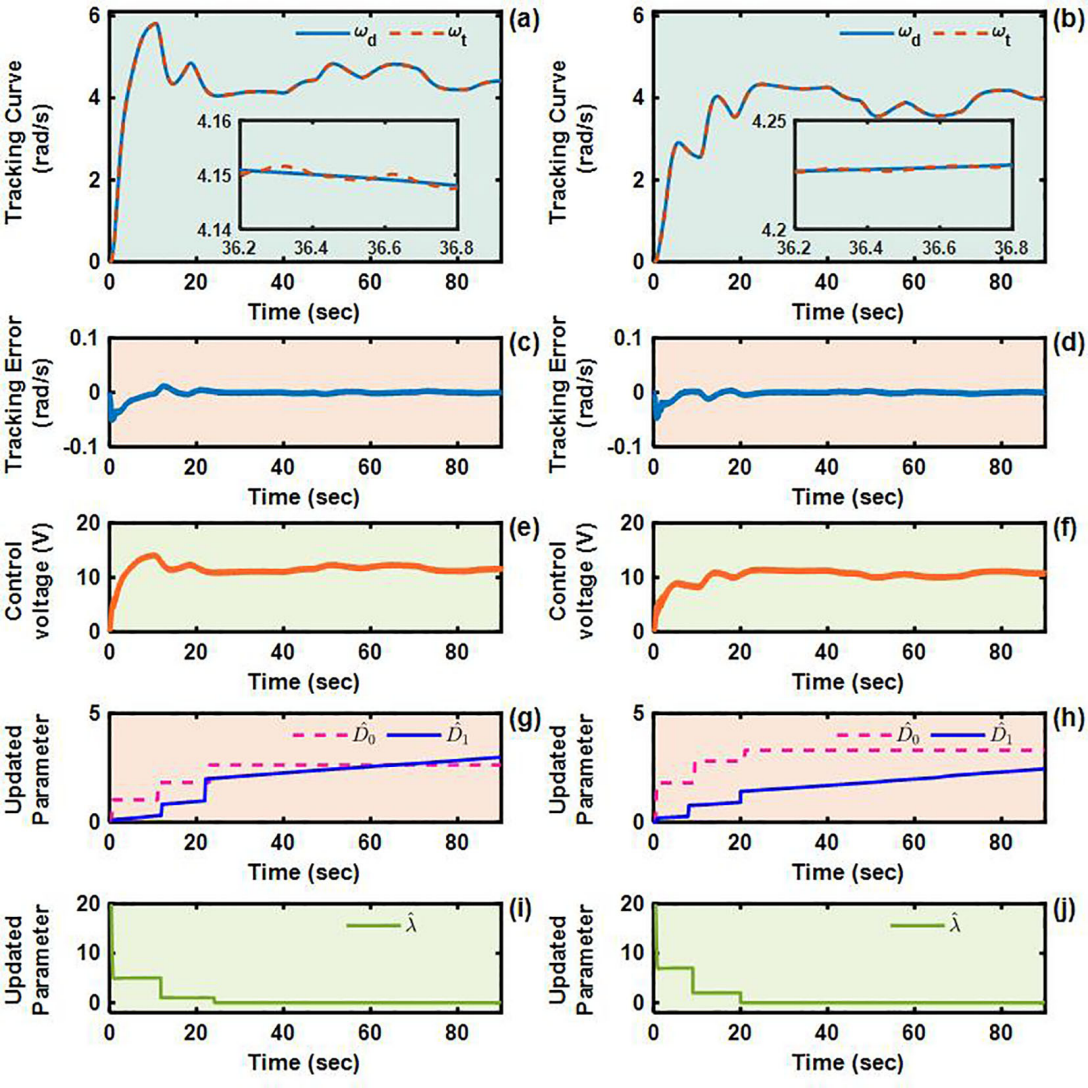
Angular velocity tracking responses of AITSM control for left and right driving wheels of TR (U-shaped path). **(a, b)** are the tracking curves of the angular velocity of left and right driving wheels. **(c, d)** are tracking errors of left and right driving wheels. **(e, f)** are control voltages of left and right driving wheels. **(g–j)** are updated parameters of left and right driving wheels.

**Figure 11:**
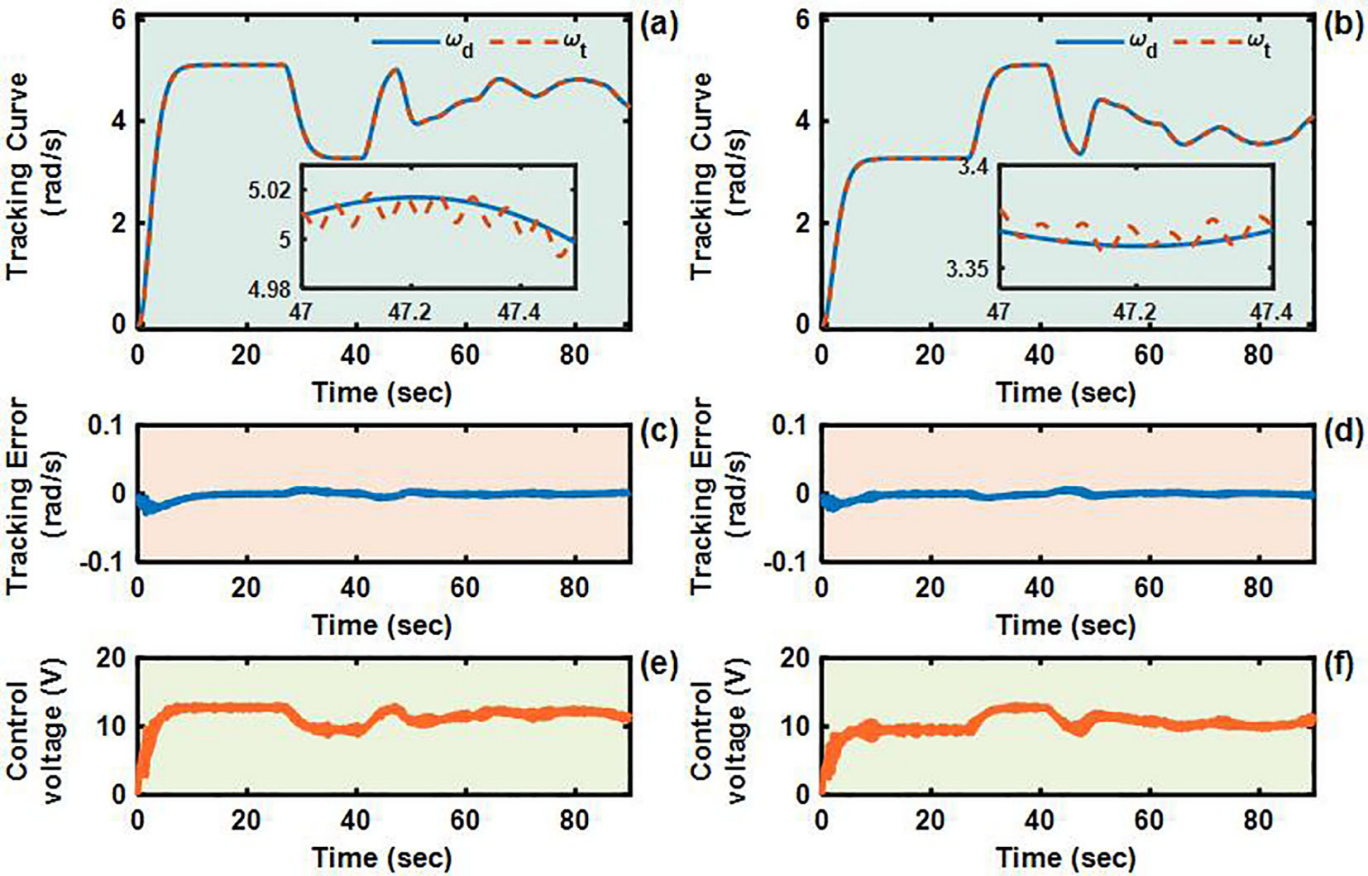
Angular velocity tracking responses of SMC control for left and right driving wheels of TR (U-shaped path). **(a, b)** are the tracking curves of the angular velocity of left and right driving wheels. **(c, d)** are tracking errors of left and right driving wheels. **(e, f)** are control voltages of left and right driving wheels.

**Figure 12:**
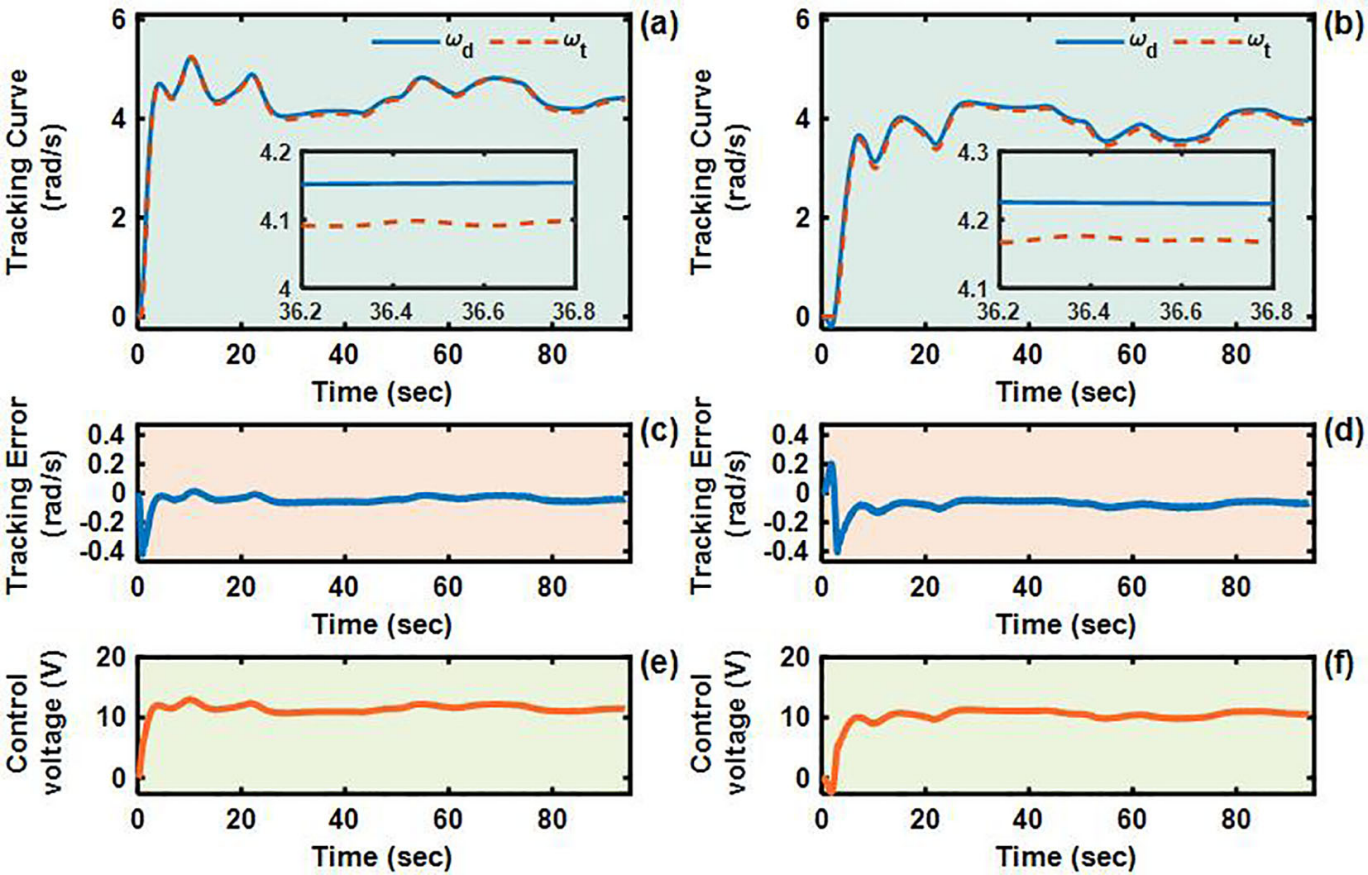
Angular velocity tracking responses of PID control for left and right driving wheels of TR (U-shaped path). **(a, b)** are the tracking curves of the angular velocity of left and right driving wheels. **(c, d)** are tracking errors of left and right driving wheels. **(e, f)** are control voltages of left and right driving wheels.

The U-shaped path tracking scenario represents a fundamental and indispensable test case for TR operating in field environments, as it accurately replicates the requirement for lines changing maneuvers while simultaneously evaluating two critical control performance aspects: the system’s ability to maintain trajectory tracking accuracy under significant soil-induced disturbances and its capacity for sustained steering control during continuous directional changes. The actual driving conditions of the TR during the U-shaped path tracking test is shown in **Figures 8**. As evidenced in **Figures 9**–**12**, the comprehensive experimental results reveal distinct performance characteristics among the proposed controllers. As shown in **Figures 9a-c**, both the proposed AITSM controller and conventional SMC demonstrate better trajectory-following capabilities with better robustness, particularly when contrasted with the PID controller which exhibits noticeable deviation, especially during the critical transition phases between straight segments and curved paths. This performance gap becomes even more pronounced when examining the drive motor angular velocity tracking responses shown in **Figures 10**-**12**. Under the demanding conditions of continuous turning, the AITSM controller maintains better steady-state performance, achieving average tracking errors of merely 0.037 rad/s and 0.021 rad/s for the left and right wheels respectively, representing a 61.8% and 80.0% improvement over the PID controller’s tracking errors of 0.097 rad/s and 0.105 rad/s. The tracking errors of the left and right drive wheels of the SMC controller are 0.029 rad/s and 0.031 rad/s respectively, and the control performance is comparable to AITSM. But SMC’s performance comes at the cost of significant high frequency chattering an inherent limitation of traditional sliding mode control architectures that arises from the discontinuous switching action required to maintain system states on the sliding surface as shown in **Figures 11e, f**. This chattering phenomenon not only persists throughout the U-shaped path maneuver but also introduces undesirable mechanical stress on actuation components, potentially compromising long term system reliability. In contrast, the AITSM controller’s adaptive control mechanisms successfully mitigate these oscillations while maintaining precision, owing to its dual layer control structure that adjusts switching gains based on real-time system. The proposed controller’s adaptive rate implementation proves effective during continuous commutation phases, as shown in **Figures 9g–j**. The experimental data further reveals that the AITSM controller’s disturbance rejection capability remains consistently effective throughout all phases of the U-shaped path maneuver, which demonstrating its adaptability to rapidly changing terrain conditions and dynamic loading scenarios. This consistent performance across field operational conditions highlights the controller’s suitability for field applications where unpredictable terrain interactions and prolonged operation requirements demand both precision and reliability.

### 4.3 Performance comparisons and discussions

For the further control performance comparisons in a quantitively way, the root means square error (RMSE) as well as the maximum error (MAXE) are used, which are defined as:


(37) $$RMSE=\sqrt{\sum_{i=1}^{N}\frac{e^{2}(i)}{N}}$$

(38) MAXE=max(|e(i)|)

where N and e(i)  represent the data samples number and the  i_th  sampled tracking error. We can see from **Table 2** that, In the L-shaped path test, the proposed controller and SMC controller is comparable, which is reflected in that the MAXE of the left and right driving wheels of the proposed controller is 17.2% and 17.0% higher than SMC respectively, but the RMSE of the left and right driving wheels of the proposed controller is 30.3% and 7.4% lower than SMC respectively. Note that, although the MAXE of proposed controller is higher than SMC controller, it appears at the initial stage of control and has little impact on the subsequent control performance, while the SMC controller, as previously mentioned, has a low MAXE but obvious chattering phenomenon. At the same time, the performance of proposed controller greatly exceeds that of the PID controller, which is reflected in that the MAXE are respectively lower by 85.9% and 88.0%, while the RMSE is respectively lower by 75.5% and 85.8%. The experimental results of the U-shaped path are similar to those of the L-shaped path. The MAXE of the left and right driving wheels of the proposed controller is 28.1% and 30.7% higher than SMC respectively, but the RMSE is 27.6% and 32.2% lower than SMC respectively. The proposed controller is 85.8% and 87.4% lower in MAXE and 61.8% and 80.0% lower in RMSE than the PID controller. By comparison, the proposed controller is superior to SMC controller and PID controller.

**Table 2:** Comparisons of control performance, unit, rad/s.

Test Case		Criteria (rad/s)	Control performance				
			Proposed controller	SMC controller	Improvement	PID controller	Improvement
Case1	**L-left**	**MAXE**	0.058	0.048	-17.2%	0.413	85.9%
		RMSE	0.023	0.033	30.3%	0.094	75.5%
	**L-right**	**MAXE**	0.047	0.039	-17.0%	0.392	88.0%
		RMSE	0.025	0.027	7.4%	0.086	70.9%
Case2	**U-left**	**MAXE**	0.057	0.041	-28.1%	0.404	85.8%
		RMSE	0.037	0.029	27.6%	0.097	61.8%
	U-right	**MAXE**	0.052	0.036	-30.7%	0.413	87.4%
		RMSE	0.021	0.031	32.2%	0.105	80.0%

## 5 Conclusion

In conclusion, this study successfully developed an Adaptive Integral Terminal Sliding Mode (AITSM) control strategy for TR operating in field environments. The experimental validation across L-shaped and U-shaped path scenarios confirmed the controller’s ability to maintain precision during dynamic maneuvers while adaptively compensating for disturbances, with tracking accuracy improved compared to PID and smoother actuation than SMC. However, the study has limitations, including the reliance on predefined disturbance models (Bekker and Janosi theories), and the need for further optimization of adaptive parameters to balance convergence speed and computational efficiency. Future research should explore the integration of machine learning techniques for disturbances identification, and investigate energy-efficient implementations for prolonged field operations.

## Data Availability

The raw data supporting the conclusions of this article will be made available by the authors, without undue reservation.
